# Long-acting relaxin analogues: a novel tool in cardiology

**DOI:** 10.3389/fphar.2025.1626469

**Published:** 2025-08-04

**Authors:** Łukasz Wołowiec, Albert Jaśniak, Joanna Osiak-Gwiazdowska, Daria Czaplińska, Agata Szymczak, Julia Anna Pęcherz, Grzegorz Grześk

**Affiliations:** Department of Cardiology and Clinical Pharmacology, Faculty of Health Sciences, Nicolaus Copernicus University in Toruń, Bydgoszcz, Poland

**Keywords:** serelaxin, relaxin, heart failure, therapeutic, pharmacology

## Abstract

Serelaxin, a recombinant human relaxin, has emerged as a potential therapy for the future treatment of heart failure. However, its effectiveness has been limited by a short half-life and the need for intravenous administration. Recently developed long-acting relaxin analogues show promise in overcoming these limitations, as they exhibit an improved pharmacokinetic and pharmacodynamic profile while preserving the beneficial actions of relaxin. Their clinical utility has been confirmed in preclinical studies as well as in recently published first-in-human, randomized study focused on heart failure treatment (study ID: NCT04630067), and improvement of renal parameters in healthy volunteers (study ID NCT04768855). In this article, we provide an overview of the mechanisms underlying the effects of long-acting relaxin analogues and their positive impact on the cardiovascular system. Additionally, we present a comprehensive comparison between serelaxin and its novel analogues, exploring their potential implications for the future treatment of cardiovascular diseases.

## 1 Introduction

Relaxin, a hormone discovered in 1926, shares structural similarities with insulin. Initially associated with reproductive functions, the cardiovascular benefits of relaxin have also been confirmed. Relaxin has been shown to exhibit dose-dependent positive chronotropic and inotropic effects on the heart (Summers et al., 2017).

Other studies have found that porcine relaxin protects the hearts of guinea pigs and rats from cardiac injury caused by ischemia and reperfusion. It improves cardiac contractility, maintains continuous coronary flow during ischemia, andenhances it during reperfusion (Masini et al., 1997; Bani et al., 1998). Furthermore, there is evidence that relaxin reduces the proliferation and differentiation of fibroblasts by inhibiting collagen secretion and stimulating an increase in matrix metalloproteinases (MMP) expression, which promotes collagen degradation, and ultimately contributes to the reduction of fibrosis (Samuel et al., 2004).

Serelaxin (the recombinant form of human relaxin-2) was initially regarded as a breakthrough therapy for acute heart failure (AHF) and has consequently been the subject of numerous preclinical and clinical trials. The primary objective of the Pre-RELAX-AHF phase II study was to evaluate the overall outcome of intravenous relaxin administration and determine the need for further investigation into the efficacy of relaxin in treating heart failure (HF) (Teerlink et al., 2009). Patients presenting with AHF and a systolic blood pressure of at least 125 mmHg, along with mild-to-moderate renal insufficiency, were eligible for enrollment in the trial (Teerlink et al., 2009). They were randomly assigned to five groups, with one receiving a placebo and the others receiving a 48-h intravenous infusion of relaxin at doses of 10 μg/kg, 30 μg/kg, 100 μg/kg, or 250 μg/kg per day (Teerlink et al., 2009). The outcomes were promising: relaxin-treated patients showed a significant improvement in dyspnoea compared to those who received the placebo (Teerlink et al., 2009). The results observed with a dosage of 30 μg/kg appeared to be the most effective (Teerlink et al., 2009). The favorable findings, particularly in terms of reducing cardiovascular mortality, underscored the need for a larger study to evaluate whether this effect is significant in a larger cohort (Teerlink et al., 2009).

In RELAX-AHF-2, patients with AHF received either a placebo or serelaxin infusion to evaluate its effects on cardiovascular mortality, with results showing similar outcomes in both groups. Unfortunately, death from cardiovascular causes at 180 days occurred at similar incidence in both groups (Metra et al., 2019).

Serelaxin, however, has a short half-life (approximately 2 h), and its effects *in vivo* diminish quickly. This is suggested to be a primary limitation in achieving long term outcomes in AHF patients (Bani, 2020). Moreover, the complex heterodimeric structure of relaxin, poses challenges in the manufacturing process (Hossain and Wade, 2014). This underscores the need for the development of new drugs that retain the cardioprotective and antifibrotic effects of serelaxin while overcoming its limitations. Long-acting human relaxin analogues have been developed to address this issue due to their reported longer duration of action, increased selectivity, higher bioavailability, and simplified structure, which makes production more cost-efficient (Hossain et al., 2016; Xiao et al., 2013; Illiano et al., 2022; Verdino et al., 2023; Muppidi et al., 2019).

Exploring the potential of long-acting relaxin analogues is crucial in the context of cardiovascular diseases. Considering the previously discussed potential benefits of relaxin regarding heart function and its remodeling effects, the development of long-acting analogues may provide invaluable support in the treatment of various diseases affecting this system, including HF. This prospect has the potential to become a powerful tool for cardiologists. In this article, we will elucidate the extensive benefits of relaxin and highlight theimportance of pioneering research into its long-acting analogues for medical practitioners, with a particular focus on its significant impact for patients.

## 2 The evolution of relaxin analogues

For many years, researchers faced challenges in obtaining well-purified relaxin extracts, leading to conflicting and inconsistent reports due to non-relaxin contaminants from ovaries, which could account for up to 90% of the sample (Summers et al., 2017). The situation improved in 1974 when relaxin was isolated in a highly purified form. That year, Sherwood and O'Byrne described both its purification and biochemical characteristics (David and O’Byrne, 1974). In the 2000s, recombinant DNA technology was developed, enabling the production of relaxin and its analogues for larger-scale research. Thanks to research on relaxin analogues, it is now possible to create molecules that are designed in such a way that, due to their structural or functional similarity, they can mimic or enhance the effects of naturally occurring relaxin. Additionally, in some cases, these molecules may possess unique properties that are intentionally tailored to achieve specific therapeutic effects (Hossain and Wade, 2014; Moore et al., 2007).

Serelaxin is a recombinant form of human relaxin-2 developed by Novartis/Corthera in Basel, Switzerland. It was produced through recombinant expression in *E. coli*, unlike wild-type human relaxin-2 and its other analogues, which were chemically synthesized using solid-phase peptide synthesis, followed by the formation of regioselective disulfide bonds or random chain combinations. Clinical development and research on serelaxin began in the late 2000s. In the RELAX-AHF study, serelaxin was found to significantly improve symptoms and signs of AHF. Unfortunately, due to the limited half-life of serelaxin, its therapeutic efficacy and the sustained benefits of its action on the cardiovascular system are limited. This has prompted subsequent studies aimed at developing analogues to extend its half-life and, consequently, enhance its therapeutic potential for patients (Illiano et al., 2022; Muppidi et al., 2019).

Previous studies have shown that the key structural site in the relaxin molecule that allows it to bind the receptor is primarily located in the alpha-helix of the B chain (Büllesbach and Schwabe, 2000; Du et al., 1982; Büllesbach et al., 1992). Fifteen relaxin B chain mimetics have been developed, but only one successfully mimicked the secondary structure of the natural ligand. Unfortunately, none of the developed mimetics was able to exert the biological activity characteristic of relaxin. This demonstrated that, in addition to the alpha-helix of the B chain, other structural elements are required to trigger a biological response when relaxin binds to its receptor. These findings were instrumental in subsequent research into new relaxin analogues (Del Borgo et al., 2005). A strategy was then undertaken to increase the potency and stability of the relaxin B chain, whichultimately led to the development of long-acting relaxin analogues. The next phase of research focused on the semi-synthetic production of these analogues. Their research led to the creation of the first innovative relaxin analogue - R9-13 (Muppidi et al., 2019). Over time, peptides from the B7-33 and B5-33 series, as well as the B10-33 series, which strongly activated the RXFP1 receptor were developed. Further improvements inmetabolite identification resulted in an even greater shortening of the peptide sequence, leading to the creation of a new series, B10-33, capable of strongly activating the RXFP1 receptor. Another advantage of these peptides was their extended half-life after intravenous administration to rats, ranging from 3 to 9 h,as well as their bioavailability following a single subcutaneous administration. The long-acting analogues described above enabled further research into their potential therapeutic use in cardiovascular diseases, particularly those associated with HF (Illiano et al., 2022).

## 3 Receptor affinity of relaxin analogues

Research conducted over the years has identified four distinct receptors, physiologically present in humans, that target peptides from the relaxin family. Each of these receptors belongs to class I receptors which are coupled to G protein. They can be divided into two groups: receptors containing C-type leucine-rich repeats: namely, RXFP1 (relaxin family peptide receptor 1) and RXFP2 (relaxin family peptide receptor 2), and those resembling receptors that regulate peptides such as angiotensin II or somatostatin: namely, RXFP3 (relaxin family peptide receptor 3) and RXFP4 (relaxin family peptide receptor 4) (Bathgate et al., 2005).

Regarding the first two receptors, RXFP1 and RXFP2, whose ligands are relaxin and INSL3 (insulin-like growth factor 3), respectively, their activation induces an increase in intracellular cAMPlevels. Furthermore, evidence suggests that RXFP1 can activate the synthesis of nitric oxide and Erk1/2 (extracellular signal-regulated kinase), and that relaxin itself is capable of entering the cell, where it activates glucocorticoid receptors (Bathgate et al., 2005).

The properties of the RXFP3 and RXFP4 receptors differ significantly, a as their stimulation leads to the inhibition of cAMP production through coupling with the Gi protein, via a mechanism sensitive to pertussis toxin (Bathgate et al., 2005; Grześk et al., 2014).

## 4 Biochemical and molecular mechanisms underlying the effects of relaxin analogues

It has been demonstrated that relaxine diverged evolutionarily from insulin and, similarlyto insulin, is produced as a prohormone consisting of three chains: A, B and C (Hsu, 2003). The C chain is cleaved and most of the relaxin in the body exists in a heterodimeric form containing chains A and B, with one an intrachain and two interchains disulfide bonds (Bathgate et al., 2018). Relaxin is encoded by seven different genes: four from the insulin-like peptide family (INSL3, INSL4, INSL5 and INSL6) and three from the relaxin peptide family (RLN1, RLN2 and RLN3) (Hsu, 2003; Samuel et al., 2007). In contrast to relaxin, its long–acting analogue B7-33 is a single–chain peptidomimeticcompound (Hossain et al., 2016; Illiano et al., 2022). There are three isoforms to which the B chain of human relaxin can be processed *in vivo*. It has been established that relaxin binds to the B–chain portion of protein and interacts with RXFP1 at a site referred to as the leucine-rich repeat (LRR) (Mallart et al., 2021). The original B-chain isoform, B1-29, is insoluble when placed in water. To create a soluble peptide, six residues from the N-terminus of B1-29 were truncated, and four residues from B1-33 were added to the C-terminus, resulting in an increase in cationic charges. This newly designed soluble form, called B7-33, is capable of interacting with RXFP1 in a manner similar to human relaxin (Hossain et al., 2016). No activation of RXFP2 by B7-33 has been observed, indicating that this analogue binds specifically to RXFP1, although with lower affinity compared to relaxin. B7-33 is known to be more easily synthesized than endogenous relaxin and with higher efficiency (Hossain et al., 2016).

Another long acting relaxin analogue is LY3540378. Like B7-33, it is a single - chain protein consisting of a human relaxin analogue fused with an albumin-binding VHH domain, which has extended its half-life while preserving the biological activity of relaxin (Verdino et al., 2023). The structure of human relaxin in this analogue includes the B chain of relaxin (with the first amino acid deleted), linked to the N-terminus of relaxin chain A via a linker (Verdino et al., 2023). Three disulfide bonds are present in the analogue, as in naturally occurring relaxin, ensuring that both its function and structure are preserved. The single-chain human relaxin is connected to the C-terminus of the serum albumin VHH domain through a flexible linker. This design results in LY3540378 exhibiting improved pharmacokinetics compared to human relaxin. Additionally, the selectivity of LY3540378 for the human RXFP2 receptor is similar to its selectivity for the human RXFP1 receptor (Verdino et al., 2023). Furthermore, another long-acting relaxin-2 analogue, R9-13, has been developed. This analogue was designed using a semisynthetic methodology to synthesize pro-relaxin conjugated with fatty acids, resulting in an extended half-life and improved efficacy compared to human relaxin (Muppidi et al., 2019). Initially, relaxin R3 was produced with a cysteine substitution at position 29 in the B-chain (replacing Ser29), followed by the deletion of the C chain. The next step involved the conjugation of fatty acids and enzymatic processing. The relaxin protein portion of the R9-13 analogue consists of two chains A and B maintaining their three natural disulfide bonds, and is bound to a fatty acid to enhance its pharmacokinetics when compared to short-acting analogues and human relaxin (Muppidi et al., 2019).

Furthermore, another notable long-acting relaxin analogue is SA10SC-RLX. It is an RXFP1 agonist that can be administered subcutaneously on a daily basis in patients. SA10SC-RLX consists of single B chain of human relaxin, linked to a PEG2 spacer and lipid - fatty acid group to enhance binding to albumins and extend its half-life (Illiano et al., 2022). SA10SC-RLX binds to RXFP1 similarly to relaxin with new interaction involving Trp28. This amino acid is stabilized by a salt bridge between Glu23 and Arg31 which strengthens the helical structure of the linker, a feature not observed in human relaxin (Illiano et al., 2022). ML290 is another compound under investigation in relaxin analogues studies. It shows selectivity for RXFP1 and is easier to synthesize than human relaxin, while maintaining similar efficacy (Xiao et al., 2013; Kaftanovskaya et al., 2019; Ng et al., 2019). ML290 is a small molecule, biased agonist at RXFP1, characterized by high stability and an improved half-life (Kocan et al., 2017). No competition between ML290 and human relaxin has been observed; on the contrary, it is believed to have an allosteric effect as the combination of both leads to an increase in binding affinity for RXFP1 (Kocan et al., 2017; Hu et al., 2016). Studies have demonstrated that ML290 occupies the ligand-binding pocket of RXFP1 with van der Waals and hydrophobic interactions occurring between residues TM5 and TM7,which are part of the receptor’s transmembrane exoloops. No activity has been observed for ML290 on RXFP2 (Kocan et al., 2017; Hu et al., 2016).

### 4.1 Cellular responses in cardiovascular tissues to long-acting relaxin analogues

Considering the cellular responses induced by long-acting analogues in cardiovascular tissues, one example is B7-33. Since it consists of only one B-chain, it is a weaker agonist of RXFP1 compared to serelaxin, a short-acting relaxin analogue (Marshall et al., 2017). Studies have shown that B7-33 can enhance bradykinin-mediated relaxation in the abdominal aorta and mesenteric arteries, but is unable to activate acetylcholine-mediated relaxation in the same manner as relaxin (Marshall et al., 2017). B7-33 is not believed to mediate an nitric oxide (NO)-dependent pathway, but instead, it is thought to participate in endothelial-dependent relaxation, intermediating calcium- and potassium channel-dependent endothelium-derived hyperpolarizing factor (EDH) activation, similarly to human relaxin (Marshall et al., 2017). Additionally, a study demonstrated that B7-33 suppresses macrophage infiltration (Alam et al., 2023).

**FIGURE 1:**
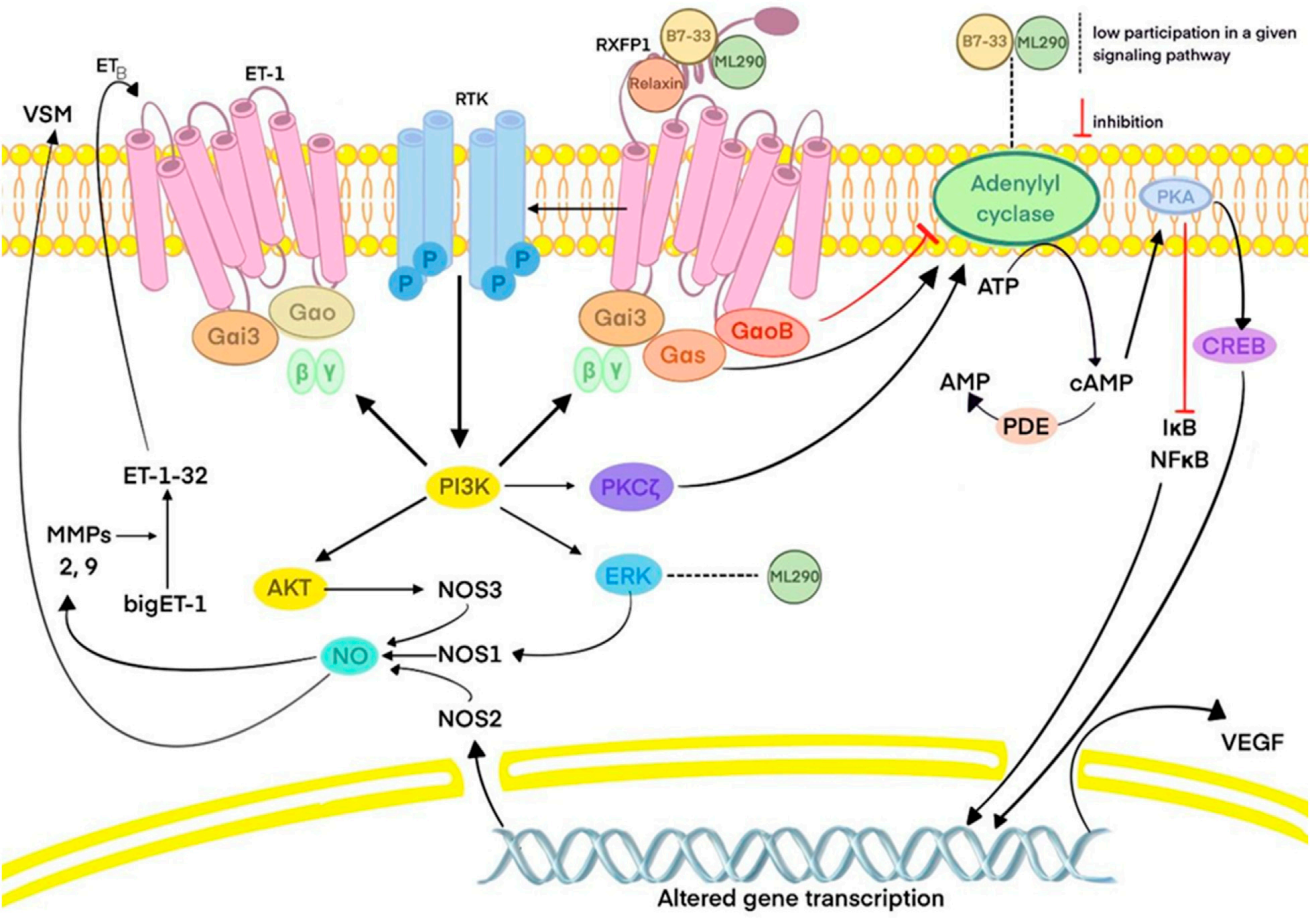
Relaxin, B7-33 and ML290 signaling pathways. Abbreviations: PKA: protein kinase A; ATP: adenosine triphosphate; AMP: adenosine monophosphate; cAMP: cyclic adenosine monophosphate; CREB: cAMP response element-binding protein; VEGF: vascular endothelial growth factor; PDE: phosphodiesterase; IκB: inhibitor of nuclear factor kappa B; NF-κB: nuclear factor kappa B; RXFP1: relaxin family peptide receptor 1; Gαi3, Gαo, Gαs, GαoB- subunits of G protein; RTK: receptor tyrosine kinase; PI3K: phosphoinositide 3-kinase; PKC: protein kinase C; ERK: extracellular signal-regulated kinase; AKT: protein kinase B; NOS: nitric oxide synthase; NO: nitric oxide; VSM: vascular smooth muscle; ET: endothelin; MMPs: matrix metalloproteinases.

**FIGURE 2:**
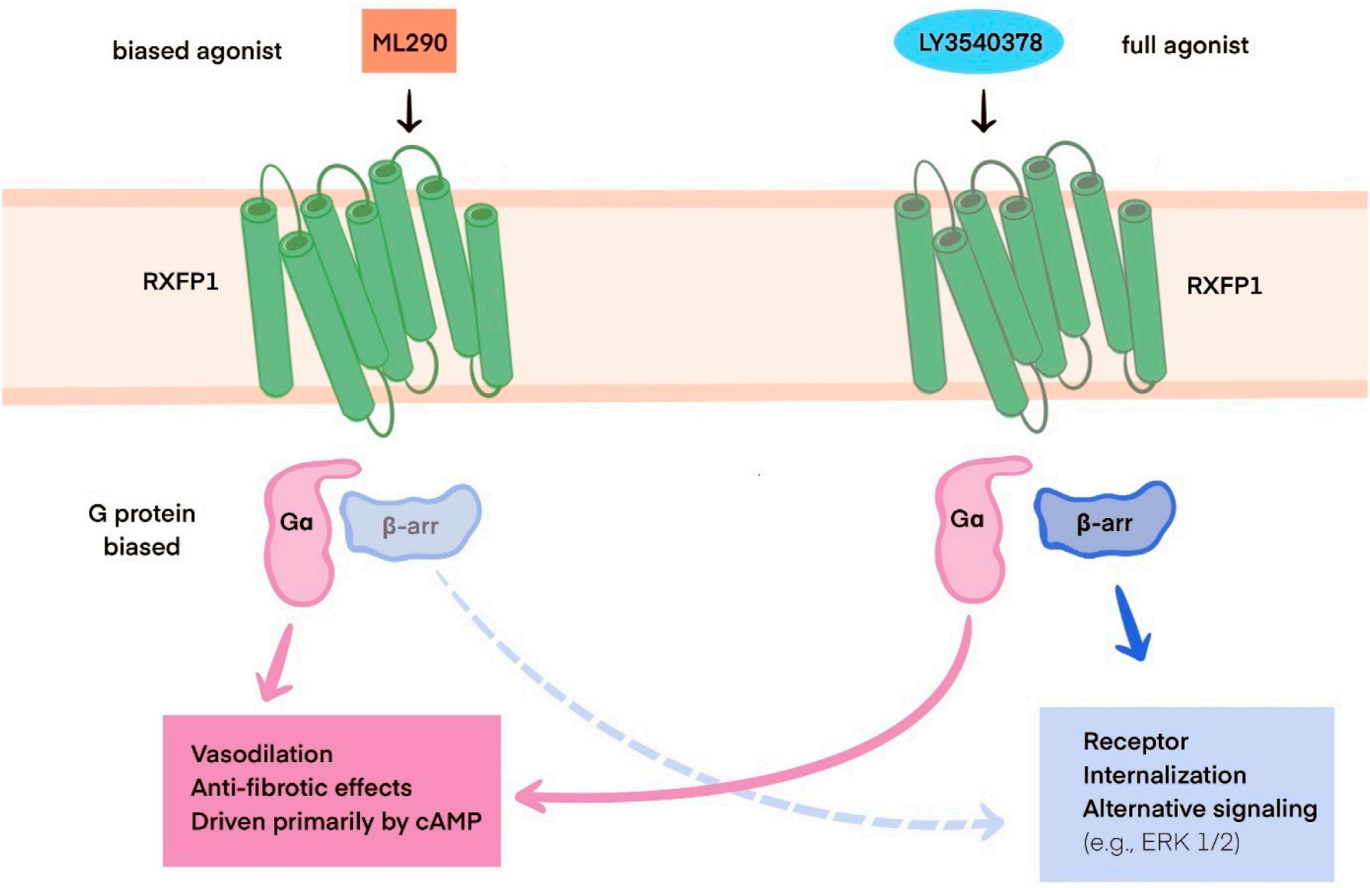
Schematic representation of the differences between RXFP1 biased agonists (e.g., ML290) and full agonists (e.g., LY3540378). Abbreviations:RXFP1: relaxin family peptide receptor 1; Gα - subunit of G protein, cAMP: cyclic adenosine monophosphate; β-arr: β-arrestin, ERK 1/2: ERK: extracellular signal-regulated kinase ½.

This process typically results in the production of TGFβ1, which leads to collagen deposition, the basis of fibrosis in cardiovascular tissues. When the TGFβ1 production is reduced, as observed with the application of B7-33, the risk of cardiac fibrosis is diminished (Alam et al., 2023). Overall, long–acting relaxin analogues as RXFP1, agonists promote pathways similar to those of relaxin as described in part IIIC. However, ML290, a biased agonist of RXFP1, induces somewhat different effects, as previously described, due to its allosteric activity when binding, in contrast to other relaxin analogues. Nevertheless, it still promotes cAMP and cGMP production but without activating the ERK1/2 pathway (Kocan et al., 2017). In contrast, LY3540378, R9-13 and SA10SC-RLX have been shown to induct cAMP production, although not at the same high levels as human relaxin (Illiano et al., 2022; Verdino et al., 2023; Muppidi et al., 2019). The relaxin, B7-33, and ML290 signaling pathways are presented in Figure 1. The differences between biased agonists and full agonists of RXFP1 are illustrated in Figure 2 using a schematic representation.

### 4.2 Comparison of mechanisms between natural relaxin and its long-acting counterparts

The binding of human relaxin-2 to RXFP1 (G protein-coupled receptor relaxin family peptide receptor 1) in cardiovascular tissues stimulates the cAMP (cyclic adenosine monophosphate), ERK (extracellular-signal-regulated kinases) and NO/cGMP pathways (Hossain et al., 2016; Valkovic et al., 2019; Summers, 2017). The NO pathway has been shown to be crucial for the homeostasis of the human cardiovascular system (Grześk et al., 2023). Furthermore the NO pathway represents a potential target for the treatment of pulmonary arterial hypertension (Wołowiec et al., 2023a), and it plays a critical role in the pathogenesis of septic shock, where NO is overproduced (Grześk and Nowaczyk, 2021). Upon RXFP1activation by relaxin biding, there is dissociation of G proteins, such as Gα which leads to an increase of cAMP levels. This activation also triggers PKA, which in turn promotes the ERK 1/2 pathway (Valkovic et al., 2019). The dissociated Gβ and γ subunits activate P3IK, thereby promoting the NO and cGMP pathways (Valkovic et al., 2019). In summary, this results in increased levels of NO, cyclic AMP and cyclic GMP. Additionally elevated levels of mitogen-activated protein kinases (MAPKs), MMPs and vascular endothelial growth factor (VEGF), an angiogenic growth factor, have been observed (Almeida-Pinto et al., 2023). These factors contribute to an anti-fibrotic effect in cardiovascular tissues, promote angiogenesis, and inhibit the fibrosis process (Wołowiec et al., 2023b). Moreover, inflammation plays a significant role in the pathogenesis of heart failure (HF), and the increased levels of MAPKs can help mitigate this pro-inflammatory response (Almeida-Pinto et al., 2023). The MAPK pathway also exerts an anti-apoptotic effect on endothelial cells in the heart (Wołowiec et al., 2024).

Long-acting relaxin analogues are expected to retain the cardioprotective benefits of natural relaxin while overcoming its limitations, such as a short half-life and the requirement for r intravenous administration. These analogues exhibit a significantly longer duration of action and can be administered subcutaneously or orally. Moreover, in contrast to relaxin, its long-acting analogue B7-33 binds to RXFP1 with lower affinity, preferentially activating the pERK pathway, thereby exerting a lesser impact on cAMP signaling activity (Hossain et al., 2016). It is hypothesized that the strong cAMP activation associated with relaxinmay contribute to its adverse effects including excessive inotropy, mortality and the promotion of tumorigenesis observed in in vivo studies. Preliminary data suggest that B7-33, unlike natural relaxin, does not promote prostate cancer progression in a mouse model (Hossain et al., 2016). A comparison of long-acting relaxin analogues is provided in Table 1.

**TABLE 1:** Comparison of long-acting relaxin analogues.

	Receptor	Activated pathway	Structure	Administration	Estimated half life
Relaxin	RXFP1 and RXFP2	Cyclic adenosine monophosphate pathway Cyclic guanosine monophosphate pathway ERK pathway	Polipeptid chain A and B with one intra-chain and two inter-chain disulphide bonds	Intravenous	<10 min
LY3540378	RXFP1 and RXFP2	Cyclic adenosine monophosphate signaling pathway	Chain A and B of relaxin with albVHH	Subcutaneous	Rats: 36.4 h Monkeys: 45.7–124 h
ML290	RXFP1	Cyclic adenosine monophosphate pathway Cyclic guanosine monophosphate pathway	Biased non-peptidic RXFP1 agonist	Oral/intravenous	Mice: 8.56 h
SA10SC-RLX	RXFP1	Cyclic adenosine monophosphate pathway	Chain B of relaxin with fatty acid	Subcutaneous	Rats: 4 h Minipigs: 7 h
R9-13	RXFP1	Cyclic adenosine monophosphate pathway	Chain A and B of relaxin with fatty acids	Subcutaneous	Rats: 11.5 h
B7-33	RXFP1	ERK pathway rather thancyclic adenosine monophosphate pathway	Chain B of relaxin in a soluble form	Subcutaneous	∼60 min
AZD3427	RXFP1	Cyclic adenosine monophosphate pathway Cyclic guanosine monophosphate pathway ERK pathway	Fc fragment of human IgG1 and one copy of relaxin-2	subcutaneous	Monkeys: 112–120 h

Abbreviations: ERK, extracellular-signal-regulated kinases; RXFP1/RXFP2, relaxin/insulin-like family peptide receptor; albVHH, albumin binding variable heavy chain domain of a heavy-chain antibody.

## 5 Research review

Research conducted around 2010 focused on the potential use of relaxin and its analogues in cardiovascular diseases. Clinical trials were subsequently initiated to evaluate their efficacy and safety in the treatment of cardiovascular conditions using relaxin analogues.

### 5.1 Review of studies evaluating serelaxin

#### 5.1.1 Preclinical studies

The first evidence suggesting that relaxin could act as a compensatory mediator in HF in humans was provided by Dschietzig in 2001 (Dschietzig et al., 2001). He demonstrated that, as the severity of the diseaseincreases, both the concentration of relaxin in the blood and the expression of its genes in the myocardium also increase. Relaxin expression significantly increases in response to elevated pressure in the filled heart chamber and exerts a strong inhibitory effect on endothelin 1, a key mediator with constrictive effect in HF. Another important mediator in the context of HF is angiotensin II, which is also thought to be inhibited by relaxin. In this study, Dschietzig was the first to show that relaxin is produced in human cardiovascular tissues and, due to its effects on these tissues, could have a significant impact on cardiac function in individuals with HF (Dschietzig et al., 2001).

Other studies on serelaxin have shown that its administration *in vivo* for 2–5 days, or intravenously, reduces biogenic reactivity and induces a rapid improvement in vasorelaxation dependent on vessels. This response is closely related to the production of many factors of endothelial origin that have relaxant properties, including EDH, PGI2 (prostacyclin) and NO (Leo et al., 2016).

#### 5.1.2 Clinical studies

Further studies in patients with chronic, stable HF have shown that intravenous administration of relaxin leads to vasodilation, resulting in a reduction in blood pressure and an increase in cardiac stroke volume (Dschietzig et al., 2009). As research on relaxin progressedattention shifted to serelaxin, a recombinant analogue of the human peptide hormone relaxin-2, which showed promising therapeutic potential in the treatment of AHF.

One of the largest studies–RELAX-AHF - was designed to assess the relationship between 180-day mortality in patients with AHF and short-term changes in markers of organ damage and congestion. The results of this study indicated that early administration of serelaxin was associated with a reduction in 180-day mortality. Additionally, the study found that early serelaxin administration was linked to fewer symptoms of organ damage and more rapid relief from congestion during the initial days of treatment (Teerlink et al., 2013). An overview of the most important clinical studies on the use of serelaxin in the treatment of HF is summarized in Table 2.

**TABLE 2:** Overview of selected clinical trials with serelaxin on HF patients.

	Number of participants	Primary endpoint	Main outcomes
NCT00520806 Relaxin for the treatment of patients with (Pre-RELAX-AHF): a multicenter, randomized, placebo-controlled, parallel-group, dose-finding phase IIb study (Teerlink et al., 2009)	234	- Improvement in -dyspnoea measured by Likert scale and VAS; - WHF at 5 days - Renal impairment - Length of hospital stay - Days alive and out of hospital at 60 days - Death due to cardiovascular causes or readmission for heart failure or renal failure at 60 days - mortality due to cardiovascular causes at 180 days	- Improved in dyspnoea per Likert scale (p = 0.044) - Tendency towards improvement in the VAS dyspnoea (p = 0.053) - Tendency towards reduction of cardiovascular mortality or readmission due to heart or renal failure at 60 days (p = 0.053) - No significant improvement in days alive out of hospital at 60 days (p = 0.16) - No significant reduction in all-cause mortality (p = 0.17) and cardiovascular mortality (p = 0.14) at 180 days - No significant reduction of length of stay (p = 0.18) - No significant reduction of renal impairment (p = 0·19) - No significant effect on WHF at 5 days (p = 0·29)
NCT00520806 Serelaxin, recombinant human relaxin-2, for treatment of acute heart failure (RELAX-AHF): a randomized, placebo-controlled trial (Teerlink et al., 2013)	1161	- Improvement in dyspnoea measured by Likert scale and VAS AUC	- Improvement in the VAS AUC dyspnoea (p = 0.007) - Reduced cardiovascular mortality at 180 days (p = 0.028) - Reduced all-cause mortality at 180 days (p = 0.02) - Reduction of WHF at 5 days (p =0009) - Reduction of length of stay (p = 0.039) - No significant improvement in dyspnoea per Likert scale (p = 0.07) - No significant reduction of cardiovascular death or readmission to hospital for HF or renal failure (0=0.89) - No significant improvement in days alive out of the hospital up to day 60 (p = 0.2930)
NCT01870778 A multicenter, randomized, double-blind, placebo-controlled phase III study to evaluate the efficacy, safety and tolerability of serelaxin when added to standard therapy in acute heart failure patients (RELAX-AHF-2) (Metra et al., 2019)	6545	- Death from cardiovascular causes at 180 days - WHF at 5 days	- No significant reduction in cardiovascular mortality at 180 days (p = 0.77) - No significant reduction of WHF at 5 days (p = 0.19) - Similar all-cause mortality at 180 days in both groups - Similar readmission due to heart or renal failure at 180 days in both groups - Similar length of stay in both groups

Abbreviations: AUC, area under the curve; HF, heart failure; VAS, visual analogue scale; WHF, worsening heart failure.

#### 5.1.3 Causes of serelaxintrial failures and implications for future research on long-acting relaxinanalogs

Discrepancies between the results of the RELAX-AHF (Teerlink et al., 2013) and RELAX-AHF-2 (Metra et al., 2019) trials have prompted discussions regarding the reasons for serelaxin’s failure to meet primary endpoints in the latter study, as well as the broader implications for future research on relaxin analogs. Below, we present potential factors contributing to these failures and draw conclusions to inform the design of future investigations. The study design and choice of endpoints warrant particular attention. The phase III RELAX-AHF trial, involving 1,161 patients, demonstrated that serelaxin significantly improved dyspnea and reduced 180-day cardiovascular and all-cause mortality, despite not being designed to assess mortality as a primary endpoint. In contrast, RELAX-AHF-2 - a larger trial enrolling approximately 6,600 patients - was intended to confirm these mortality benefits and improve short-term heart failure symptoms over 5 days. However, the study failed to meet both primary endpoints: 180-day cardiovascular mortality (8.7% vs. 8.9% for placebo; p = 0.77) and worsening heart failure (WHF) during hospitalization (6.9% vs. 7.7%; p = 0.19). Designating mortality as a primary endpoint in RELAX-AHF-2 may have been overly ambitious, given that mortality outcomes in AHF are influenced by numerous variables, including comorbidities and non-modifiable factors such as socioeconomic status, which may have diluted any treatment effect of serelaxin. Another limitation was the potential heterogeneity of the study population. Differences in patient characteristics between the two studies may have contributed to the divergent findings. RELAX-AHF enrolled patients with specific clinical features, such as dyspnea, peripheral edema, elevated natriuretic peptide levels, mild to moderate renal dysfunction, and systolic blood pressure >125 mmHg. Although RELAX-AHF-2 employed similar inclusion criteria, subtle differences in patient demographics - such as age, prevalence of comorbid conditions (e.g., 54% had coronary artery disease in RELAX-AHF-2), and background therapies - could have influenced the outcomes. Subgroup analyses from RELAX-AHF suggested greater mortality benefit in select populations (e.g., patients aged ≥75 years or with eGFR <50 mL/min/m^2^), indicating that serelaxin’s efficacy may be population-specific. Broader inclusion criteria in RELAX-AHF-2 may have diluted these effects in less responsive subgroups. Mechanistic and pharmacodynamic limitations should also be considered. Serelaxin’s pleiotropic effects - vasodilation, anti-inflammatory activity, and organ protection (Bani, 2020) - were thought to contribute to its clinical benefits in AHF. In RELAX-AHF, serelaxin reduced biomarkers of cardiac, renal, and hepatic injury. However, in RELAX-AHF-2, no significant changes were observed in key biomarkers (e.g., no sustained reduction in high-sensitivity troponin T at 48 h), suggesting that pharmacodynamic effects may not have been consistently achieved in the larger and more heterogeneous population. Moreover, the 48-h infusion duration may have been insufficient to yield long-term clinical benefits in a complex condition such as AHF. Statistical power and event rates in the respective trials also raise concerns. The mortality reduction observed in RELAX-AHF (a 37% relative reduction in cardiovascular mortality) was based on a relatively small number of events (107 deaths, 88 of cardiovascular origin), which may have overestimated the treatment effect due to chance. RELAX-AHF-2, although involving a larger sample size, observed a lower-than-expected event rate, potentially limiting the power to detect a meaningful difference. This discrepancy suggests that the promising results from RELAX-AHF may have been a statistical artifact, underscoring the necessity of adequately powered trials to validate preliminary findings. Advances in standard-of-care therapy may have also contributed to the lack of observed benefit. Improvements in AHF management between the two studies may have attenuated the relative benefit of serelaxin. In RELAX-AHF-2, patients received optimized background therapies (e.g., 99.5% received loop diuretics, 69% beta-blockers), which could have minimized any additional effect of serelaxin compared to placebo. This highlights the inherent challenge of demonstrating efficacy against the backdrop of evolving standards of care. Future research should focus on identifying patient subgroups most likely to benefit from relaxin analogs, such as individuals with specific biomarker profiles (Wołowiec et al., 2024; Wołowiec et al., 2023c) (e.g., elevated natriuretic peptides or reduced eGFR). Biomarker-enrichment strategies could enhance the sensitivity of trials and more effectively target populations with a higher likelihood of therapeutic response. Additionally, dose optimization studies are necessary to balance efficacy with the potential immunogenic risk associated with chemically modified analogs. Detailed assessment of tumorigenic potential in chronic toxicity studies is also warranted (Kotwal et al., 2024; Kwantwi, 2023). The discrepancies between RELAX-AHF and RELAX-AHF-2 highlight the complexity of translating promising phase III results into definitive clinical benefits. Factors such as trial design, patient heterogeneity, mechanistic limitations, statistical issues, and improvements in standard therapy may all have contributed to the failure of RELAX-AHF-2. Future research on relaxin analogs should prioritize precise patient selection, optimized dosing regimens, robust mechanistic evaluations, alternative endpoints, and the development of new analogs with improved safety profiles. These strategies will be critical to unlocking the therapeutic potential of relaxin in AHF and other disease contexts.

### 5.2 Preclinical studies evaluating long-acting relaxin analogues

#### 5.2.1 Overview of animal studies assessing the efficacy and safety of long-acting relaxin analogues

The first small-molecule agonist of RXFP1, ML290, is stable both *in vivo* and *in vitro*, potent, and selective (Wilson et al., 2018). Its efficacy is comparable to that of natural relaxin (Wilson et al., 2018). When administered intravenously, ML290 exhibits a half-life of 6.6 h in mice plasma and 6.3 h in the heart. In contrast oral administration results in a half-life of 5.5 h in plasma and 1.0 h in the heart (Wilson et al., 2018). Notably, the exposure in the heart is approximately six times higher with intravenous dosing, suggesting that this route of administration may be more suitable for chronic diseases associated with fibrosis (Wilson et al., 2018). No toxicity or abnormal behavior has been reported to date (Xiao et al., 2013; Kaftanovskaya et al., 2019; Wilson et al., 2018).

B7-33 is predicted to exhibit a half-life similar to that of H2 relaxin, or potentially even shorter, due to its similar structure (Praveen et al., 2023). Its degradation in human serum *in vitro* occurs within minutes (Wilson et al., 2018). Compared to H2 relaxin, B7-33 binds to RXFP1 with lower affinity (Hossain et al., 2016). Unfortunately, B7-33 exhibits low solubility in pH 7.4 buffer and instability in plasma (Mallart et al., 2021). However, B7-33 effectively stimulates the phosphorylation of ERK1/2 and MMP (Hossain et al., 2016). Its efficacy and potency are comparable to H2 relaxin in multiple functional assays, including, among others, three different preclinical rodent models of lung and heart fibrosis (Hossain et al., 2016). Yet, B7-33 failed to induce a significant increase in cAMP, a characteristic feature of the relaxin mechanism of action (Hossain et al., 2016; D’Ercole et al., 2022). It is suggested that, due to its distinct signaling pathway, B7-33 may not induce some of the adverse effects associated with H2 relaxin, including chronotropy, inotropy, and mortality (Hossain et al., 2016; Alam et al., 2023). In contrast to serelaxin, B7-33 did not promote prostate tumor growth in mice in vivosuggesting an absence of proliferative activity (Hossain et al., 2016). Therefore, B7-33 may represent a safer alternative to SA10SC-RLX (Alam et al., 2023).

R9-13 exhibited improved efficacy *in vivo* and a half-life *in vivo* compared to serelaxin (Muppidi et al., 2019). Enhanced properties of R9-13 compared to serelaxin suggest the possibility of its subcutaneous administration once a week (Muppidi et al., 2019).

One of the most recent long-acting relaxin analogs to emerge in recent years is SA10SC-RLX, which stimulates the RXFP1 receptor. It is suitable for subcutaneous administration to patients once daily and has potential applications in the treatment of cardiovascular diseases and chronic fibrotic diseases (Illiano et al., 2022). Bioavailability of SA10SC-RLX administered subcutaneously was estimated to be close to 70% in minipigs (Illiano et al., 2022). SA10SC-RLX possesses a longer half-life and activity in plasma than relaxin. Moreover, it is proposed to be suitable for once-daily subcutaneous administration (Illiano et al., 2022).

LY3540378 demonstrated significantly higher functional potency (EC50 values) compared to native human relaxin-2 at RFXP1 receptors (Verdino et al., 2023). The analogue exhibits an excellent long half-life of 36.4 h in rats and 45.7–124 h in monkeys (Verdino et al., 2023). Higher bioavailability was achieved with subcutaneous administration as opposed to intravenous administration in both rats and monkeys (Verdino et al., 2023). The single-dose cardiovascular safety of LY3540378 was tested in monkeys (Verdino et al., 2023). It was observed to cause an increase in heart rate, the rate of rise in left ventricular pressure, and a decrease in blood pressure (Verdino et al., 2023). However, these effects were transient, which does not preclude the potential utility of LY3540378 in chronic treatment (Verdino et al., 2023).

In 2025, a study was published investigating the long-acting relaxin analog AZD3427 in non-human primates with left ventricular systolic dysfunction and metabolic syndrome. AZD3427 was administered subcutaneously once a week for a period of 21 weeks, during which significant improvements were observed in cardiac function, including ejection fraction (EF), cardiac output (CO), and stroke volume (SV), as well as a reduction in systemic vascular resistance (SVR). The researchers reported no adverse events related to the treatment, nor any associated changes in heart rate (HR) or blood pressure (BP). During the 18-week washout period, the observed cardiovascular benefits gradually diminished (Connolly et al., 2024)

### 5.3 Insights into the cardiovascular benefits observed in preclinical models

ML290 is suggested to exhibit vasodilatory effects while simultaneously preventing hypertrophy and angiogenesis (Kocan et al., 2017). However, no research has yet evaluated the outcomes of treatment with ML290 in cardiovascular diseases. Nonetheless, ML290 has demonstrated antifibrotic properties in liver fibrosis, as well as anti-apoptotic and extracellular matrix remodeling properties in the kidney (Kaftanovskaya et al., 2019; Wilson et al., 2018; Ng et al., 2021; Bhuiyan et al., 2021).

B7-33 has been shown to exhibit multiple cardioprotective effects similar to those observed with serelaxin. Systemic administration of B7-33 following myocardial infarction (MI) in rats resulted in a reduction in collagen accumulation in the left ventricle (LV), thereby improving LV function (Hossain et al., 2016). B7-33 treatment, administered after ischemia-reperfusion injury in mice, led to a significant reduction in infarct size, preservation of cardiac function 24 h post-MI, and inhibition of further adverse remodeling in the heart (Devarakonda et al., 2020). Additionally, B7-33 was found to enhance fibroblast cell survival (Devarakonda et al., 2020). Fibroblast proliferation is thought to be crucial in theearly stages of myocardial infarction healing, suggesting that B7-33 may have long-term antifibrotic effects and, therefore, potential as a novel therapy for MI (Devarakonda et al., 2020). Furthermore, B7-33 retains the beneficial vascular effects of serelaxin in mesenteric arteries of healthy male rats. Acute intravenous injection of both B7-33 and RLX led to comparable bradykinin-induced vasodilatation in the mesenteric arteriesof healthy rats but did not enhance acetylcholine-mediated relaxation (Marshall et al., 2017). The small renal artery and aorta were unaffected by both B7-33 and H2 Relaxin (Marshall et al., 2017; Alam et al., 2023). Moreover, B7-33 inhibited trophoblast conditioned media-induced endothelial dysfunction, which is characteristic of preeclampsia (Marshall et al., 2017).

The cardioprotective effects of B7-33 were further examined in mice subjected to the isoprenaline-induced model of cardiomyopathy (Alam et al., 2023). Its administration affected fibrosis by downregulation TGF-β1 expression, decreasing in interstitial LV myofibroblast accumulation, and reducing collagen deposition (Marshall et al., 2017). Moreover, B7-33 attenuated LV inflammation by reduction LV macrophage infiltration and inhibiting LV (p)-IκB levels (Alam et al., 2023). Additionally, B7-33 reduced cardiomyocyte, restored vascular rarefaction to pre-ISO-damage levels, and attenuated aortic contraction (Alam et al., 2023). Notably, B7-33 demonstrated superior clinical efficacy compared to the angiotensin-converting enzyme inhibitor (ACEI) perindopril (Alam et al., 2023). Furthermore, B7-33 shows potential as a suitable alternative to relaxin due to its broad therapeutic effect and increased selectivity (Alam et al., 2023). In summary, B7-33 replicates the anti-fibrotic, anti-inflammatory, anti-hypertrophic, angiogenic and vasoprotective effects of recombinant human relaxin (Hossain et al., 2016; Alam et al., 2023; Devarakonda et al., 2020). These findings suggest that B7-33 should be considered as a potential future anti-fibrotic and vasoactive therapy, making it suitable for a wide range of cardiovascular diseases.

Administration of R9-13 in mice resulted in pubic ligament elongation (Muppidi et al., 2019). Furthermore, this effect persisted for 1 week post-injection (Muppidi et al., 2019). SA10SC-RLX replicates the actions of relaxin in both *in vitro* and *in vivo* models (Illiano et al., 2022). It was found to increase renal plasma flow while simultaneously decreasing renal vascular resistance in rats (Illiano et al., 2022). Kidney failure is an important prognostic factor for worsening HF; therefore, SA10SC-RLX is suggested to be suitable for the treatment of acute decompensated HF (Illiano et al., 2022). LY3540378 was observed to reduce serum osmolality and increase renal blood flow in both rats and mice (Verdino et al., 2023). A single administration of LY3540378 induced a long-lasting tachycardic effect, suggesting its potential utility in chronic disorder treatment (Verdino et al., 2023). The drug elevated blood pressure while simultaneously having no effect on the glomerular filtration rate (GFR), potentially leading to decrease in filtration fraction (FF) and intraglomerular pressure (Verdino et al., 2023). The renal effect of LY3540378 may indicate its value in the treatment of volume overload in HF patients (Verdino et al., 2023). Administration of LY3540378 to mice with isoproterenol-induced cardiac hypertrophy resulted in a significant reduction in heart weight/tibia length ratio, anterior and posterior wall thickness, and isovolumic relaxation time (Verdino et al., 2023). Moreover, the use of a higher dose of LY3540378 led to an even greater attenuation of cardiac hypertrophy and improvement in diastolic function (Verdino et al., 2023).

#### 5.3.1 pharmacokinetic–Pharmacodynamic relationship and therapeutic window

Despite promising efficacy signals observed in both animal models and early-phase clinical trials, detailed pharmacokinetic–pharmacodynamic (PK/PD) modeling data for long-acting relaxin analogues remain scarce. Available preclinical studies have primarily reported on surrogate efficacy endpoints, such as reductions in systemic vascular resistance, improvements in cardiac output, or attenuation of fibrosis, without formal modeling to establish minimum effective concentrations (MEC) or maximum tolerated doses (MTD).

As previously presented in the table, the analogues differ significantly in terms of structure, receptor selectivity, and bioavailability—ranging from subcutaneous half-lives of approximately 60 min for B7-33 to over 120 h for AZD3427. However, it remains unclear whether the observed *in vivo* effects fully align with the desired therapeutic window in humans. Bridging studies defining human-equivalent dosing from animal data are currently lacking or unpublished.

Moreover, the mechanism of action—biased vs. full agonism—may influence downstream effects such as cAMP/ERK balance, yet these are seldom correlated with pharmacodynamic outputs *in vivo*. ML290, for instance, promotes cAMP accumulation without ERK activation, which might confer a more favorable safety profile, but the clinical relevance of this bias remains to be validated (Xiao et al., 2013; Kaftanovskaya et al., 2019; Kocan et al., 2017).

Given the structural heterogeneity of these compounds (peptides, small molecules, Fc-fusion proteins), and their variable half-lives and signaling profiles, consistent PK/PD frameworks are essential for proper dose optimization. As emphasized by Verdinoet al. And Jelinic et al., more comprehensive human data are needed to clearly define the exposure–response relationship and establish clinical margins of efficacy and safety. This represents a key translational challenge for the field (Jelinic et al., 2018).

## 6 Clinical trials

**FIGURE 3:**
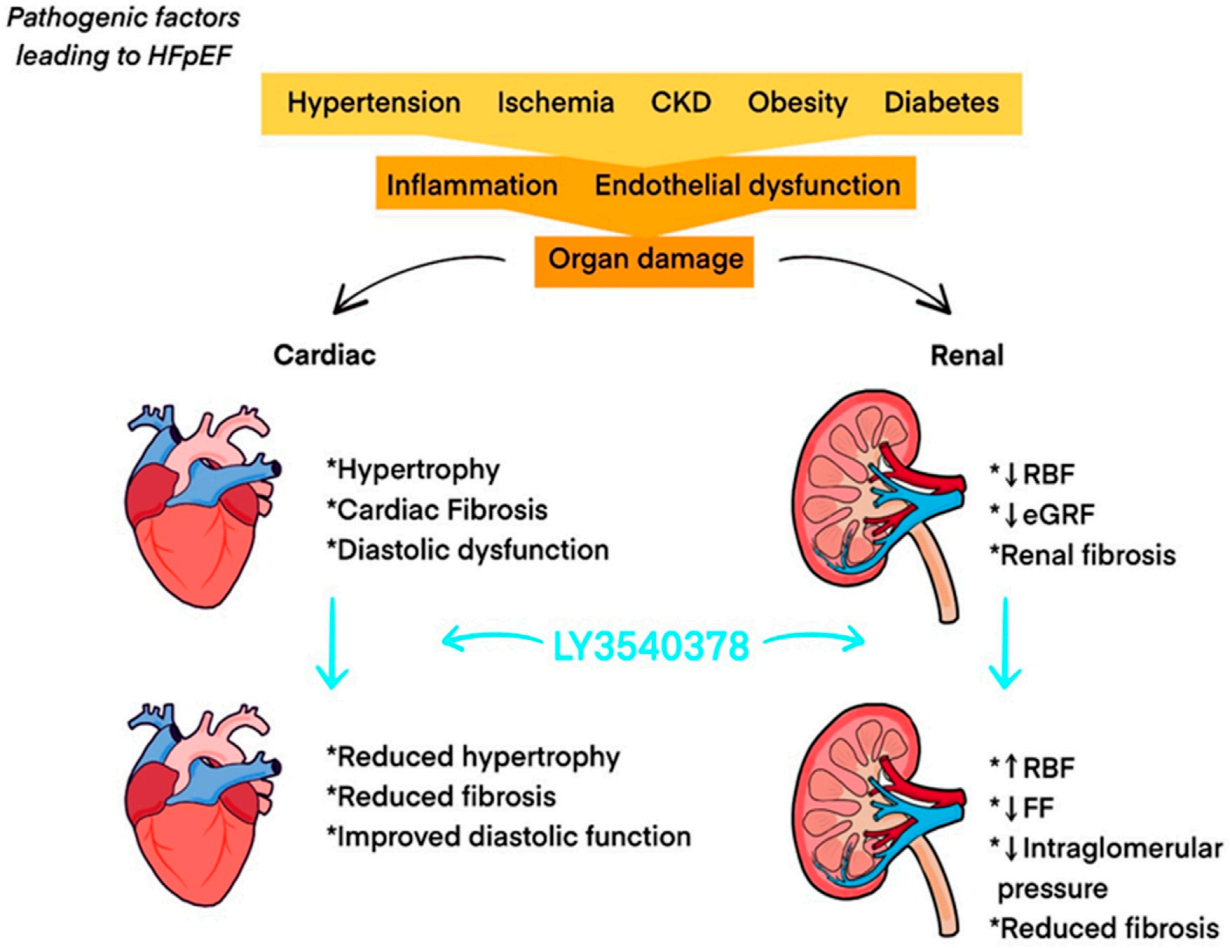
Pathophysiology of HFpEF and effects of LY3540378. Abbreviations: CKD: chronic kidney disease; eGFR: estimated glomerular filtration rate; FF: filtration fraction; HFpEF: heart failure with preserved ejection fraction; RBF: renal blood flow.

LY3540378 has completed a Phase 2 study aimed at evaluating its efficacy and safety in adults with worsening chronic heart failure with preserved ejection fraction (HFpEF) (Eli Lilly and Company, 2022). The study included 335 patients, who were randomized into placebo and treatment groups, receiving various doses of LY3540378 (Eli Lilly and Company, 2022). The primary endpoint is the change from baseline in in left atrial reservoir strain (LARS) (Eli Lilly and Company, 2022). The results of this trial are highly anticipated, as they may provide evidence of the therapeutic potential of long-acting relaxin analogues and introduce much-needed new treatments for chronic HF. Unfortunately, despite significant progress in the treatment of HF over the past few decades, the efficacy of therapy for HFpEF has not improved (Reddy and Borlaug, 2016). The prevalence of HFpEF continues to rise, underscoring the urgency of advancing treatment options for this disorder (Tsao et al., 2018). The pathophysiology of HFpEF and the effects of LY3540378 are illustrated in Figure 3. Another recently published clinical study, conducted in August 2024, investigates the RXFP1 relaxin receptor agonist AZD3427 in the treatment of heart failure. Although limited by the small number of participants with heart failure (n = 48), the study shows an increase in stroke volume and estimated glomerular filtration rate in the study group, which is consistent with the expected effects of relaxin analogues (Connolly et al., 2024).

Furthermore, in a Phase I clinical trial involving healthy participants, LY3540378 was shown to be safe, effective, and well-tolerated with chronic dosing across a broad range of doses. The treatment resulted in improvements in effective plasma flow through the kidneys, filtration fraction, and renal vascular resistance with weekly repeated dosing (Tham et al., 2024). The pleiotropic effects of LY3540378 hold promise as a novel therapeutic approach. A review of selected clinical studies on the use of long-acting relaxin analogues in the treatment of cardiovascular diseases is presented in Table 3.

**TABLE 3:** Review of selected clinical studies on long-acting relaxin analogues.

parameter	NCT04630067 (Connolly et al., 2024)	NCT05592275 (ClinicalTrials.gov, 2023)	NCT04768855 (Tham et al., 2024)	NCT06616974 (ClinicalTrials.gov, 2024)
Type of study	A Phase 1a/b, First-in-Human, Randomized, Single-Blind, Placebo-Controlled Study	A Phase 2, Randomized, Double-Blind, Placebo-Controlled Study	A Phase 1, four-part, randomized, double-blinded, placebo-controlled Study	A Phase 2double-blind, randomized, parallel group, placebo-controlled, proof- of-concept
Purpose of the study	Safety and pharmacokinetic assessment	Safety and effectiveness assessment	Safety, tolerability and pharmacokinetic assessment	Safety and effectiveness assessment
Status	Completed	Terminated	Completed	Recruiting
Molecule	AZD3427	Volenrelaxin (LY3540378)	Volenrelaxin (LY3540378)	Fc-relaxin fusion protein (TX000045, TX45)
Dosage	5, 15 or 45 mg given weekly for 5 weeks	Not published yet	Single- and multiple-ascending doses given weekly for 5 weeks	Single dose A given every 2 weeks, or single dose B alternating with placebo every 2 weeks for 24 weeks
Drug administration	s.c	s.c	s.c, i.v	s.c
Population	HFrEF HFmrEF HFpEF	Exacerbated HFpEF	Healthy volunteers	Pulmonary Hypertension secondary to HFpEF
Number of participants	105	335	134	180
Clinical trial period	2020.11.17–2022.09.14	2023.02.03 – 2025.01.22	2021.03.17–2022.05.30	2024.10.08–2026.11.20
Results	Favorable safety and pharmacokinetic profiles ↑SV, eGFR ↓SBP, DBP	Not published yet	favorable safety and pharmacokinetic profiles, no increase in orthostatic hypotension sustained improvement in kidney perfusion (ERPF increased from baseline, acute and chronic increase observed) ↓SBP, DBP	Not published yet

Abbreviations: HFrEF, heart failure with reduced ejection fraction; HFmrEF, heart failure with mildly reduced ejection fraction, HFrEF, heart failure with reduced ejection fraction, SV, stroke volume, eGFR, estimated glomerular filtration rate, ERPF, effective renal plasma flow; s. c, subcutaneousi; v, intravenous.

## 7 Comparative analysis

### 7.1 Comparison of long-acting relaxin analogues with traditional relaxin therapies

Relaxin’s complex structure makes its production costly, whereas long-acting analogues offer a simplified and more affordable alternative (Hossain et al., 2016; Marshall et al., 2017). Advancements in the understanding of structure–activity relationships (SARs) have facilitated the development of novel RXFP1 agonists (Hossain and Wade, 2014; Hossain and Bathgate, 2018). The simplified structure of long-acting relaxin analogues, compared to serelaxinreducues synthesis costs, enabling the possibility of conducting preclinical and clinical evaluations on a large scale. This also increases the drug’s afford ability and accessibility to a broader patient population (Hossain et al., 2016; Xiao et al., 2013; Verdino et al., 2023). The longer half-life of these analogues may result in a more favorable pharmacokinetic profile for chronic use. LY3540378 is expected to maintain a more stable and sustained level in the bloodstream, mimicking the exposure to relaxin observed during pregnancy (Verdino et al., 2023). The disappointing results of clinical trials in which serelaxin was administered for 48 h highlight the need to extendthe duration of the treatment (Almeida-Pinto et al., 2023). Due to the short half-life of serelaxin, repeated infusions are necessary for effective HF treatment (Almeida-Pinto et al., 2023). The intravenous route of serelaxin administration reflects its typical use in hospital settings.

Long-acting relaxin analogues such as B7-33, LY3540378, SA10SC-RLX, administered subcutaneously, demonstrate high efficacy (Hossain et al., 2016; Xiao et al., 2013; Illiano et al., 2022; Verdino et al., 2023; Muppidi et al., 2019). This method of administrationallows the drug to be given at home, eliminating the need for hospitalization. The greater stability of long-acting relaxin analogues leads to less frequent dosing, without compromising the therapeutic efficacy. Consequently, this contributes to improved patient compliance, underscoring the suitability of long-acting relaxin analogues for long-term chronic treatment. It is widely accepted that oral delivery would be the most convenient form for patients. Unfortunately, ML290, which was initially proposed to be orally available failed to replicate the same half-life in the mouse heart that was observed with intravenous administration (Wilson et al., 2018). Its nearly six fold lower availability in the heart compared to plasmaraises concerns about the clinical utility of its oral administration for cardiovascular fibrosis-related diseases including HF (Wilson et al., 2018). No immunological adverse reactions to serelaxin have been reported to date. However, circulating anti-RLX antibodies have been detected in approximately 20% of patients with scleroderma who were treated with the drug (Seibold et al., 2000). The presence of these antibodies may lead to partial inactivation of serelaxin, potentially reducing its efficacy. ML290, as a biased allosteric agonist, bypasses this issue of immune response (Kaftanovskaya et al., 2019).

**FIGURE 4:**
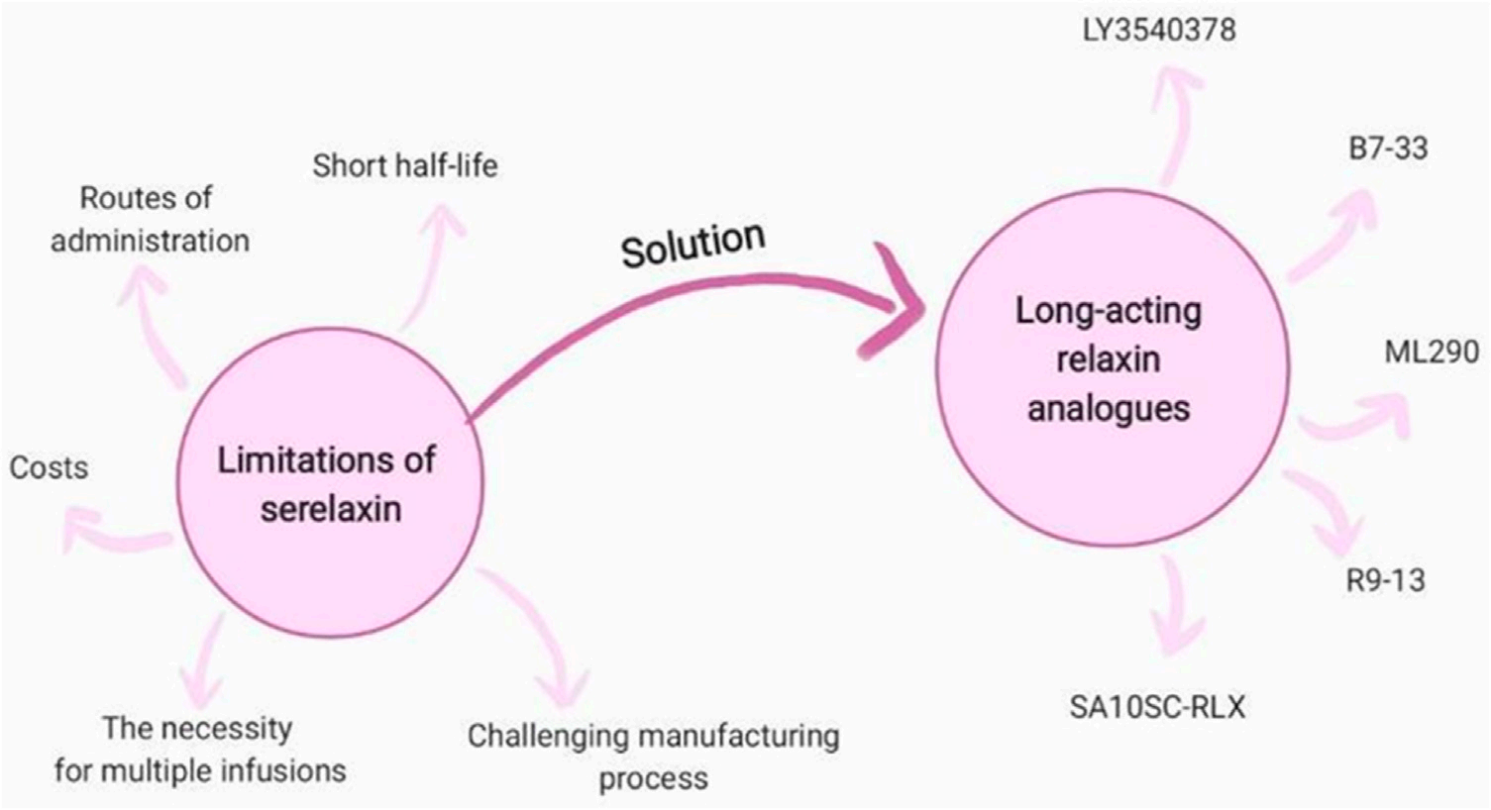
Long-acting relaxin analogues as a solution for limitations of serelaxin.

Recombinant relaxin is generally well tolerated, with no major adverse effects observed in clinical trials of serelaxin (Teerlink et al., 2020). However, potential adverse reactions associated with long-acting relaxin analogues, if identified, could limit their clinical utility. Phase 1 clinical trial results for AZD3427 (Connolly et al., 2024) and LY3540378 (Tham et al., 2024) highlight a favorable safety profile and good treatment tolerability. The most commonly reported adverse events were localized injection site reactions, such as erythema and swelling. One participant was withdrawn from the NCT04768855 study due to elevated alanine aminotransferase activity (Tham et al., 2024). A single serious adverse event-pericarditis - was observed in the placebo group (Tham et al., 2024). No treatment-emergent anti-drug antibodies were reported during the course of therapy (Connolly et al., 2024). The main limitations of serelaxin are summarized in Figure 4.

## 8 Potential barriers to clinical adaptation of long-acting relaxin analogues

The role of relaxin, relaxin-like proteins, and RXFP receptors in tumorigenesis remains unclear (Thanasupawat et al., 2019; Kotwal et al., 2024; Kwantwi, 2023). Research suggests that RLN peptide receptor signaling may exert pro-tumorigenic or anti-tumorigenic effects depending on the cancer type (Rizvi and Gores, 2020; Kotwal et al., 2024). Expression of RLN-2 in cancer is associated not only with an aggressive phenotype and metastatic potential but may also serve as a prognostic factor (Kwantwi, 2023). Pro-tumorigenic effects may arise from mechanisms such as direct stimulation of proliferation through the endogenous RLN-RXFP1 axis and indirect effects, including induction of M2 macrophages, angiogenesis, and extracellular matrix (ECM) degradation via promotion ofMMPs, which may facilitate tumor invasion. Conversely, enhanced MMP-mediated degradation may reduce tumor progression by improving the penetration of chemotherapeutic agents. Direct interactions of RLN with tumors may also exert anti-tumorigenic effects, potentially through enhanced infiltration of cytotoxic T lymphocytes, reduced activation of cancer-associated fibroblasts, and inhibition of metastasis via increased E-cadherin synthesis (Kotwal et al., 2024; Kwantwi, 2023). Despite significant concerns and potential limitations in the use of long-acting relaxin analogs, there is a paucity of studies addressing their safety profile in the context of tumorigenesis. For instance, a single *in vivo* study reported no prostate cancer progression under the influence of B7-33, in contrast to serelaxin. Given the dual role of relaxin in tumorigenesis, the biological mechanisms underlying relaxin’s action in carcinogenesis remain unclear. Future studies on long-acting relaxin analogs should focus on their multifaceted roles in carcinogenesis. Long-acting relaxin analogs, particularly those that are chemically modified (e.g., pegylated peptides or fusion proteins), may elicit an immune response that can progressively reduce therapeutic efficacy or lead to adverse effects. Potential immunological risks include the development of neutralizing antibodies that diminish the biological activity of the drug, hypersensitivity reactions -including anaphylactoid or inflammatory responses - and long-term consequences of immune system stimulation, which remain poorly understood in the context of relaxin-based therapies. The requirement for long-term immunotoxicological studies and ongoing patient monitoring to assess immunogenicity may delay regulatory approval and limit clinician confidence in this new class of therapeutics. Relaxin is a structurally complex peptide hormone, and its long-acting analogs often necessitate advanced protein engineering techniques, such as the synthesis of dual-chain constructs with correct disulfide bond formation and structural modifications (e.g., pegylation or Fc-fusion with immunoglobulin G fragments). These modifications increase the complexity and cost of manufacturing and demand highly controlled production environments (e.g., compliance with current Good Manufacturing Practices, sterility, and stringent control of protein aggregation). The production of long-acting relaxin analogs is therefore more resource-intensive and time-consuming than that of traditional small-molecule drugs, which may limit the scalability and accessibility of such therapies. In recent years, the management of HF has markedly improved due to the availability of inexpensive and effective therapies, such as ACE inhibitors (ACEIs), angiotensin receptor blockers (ARBs), angiotensin receptor–neprilysin inhibitors (ARNIs), mineralocorticoid receptor antagonists (MRAs), and sodium-glucose co-transporter 2 (SGLT2) inhibitors. These treatments are supported by robust evidence demonstrating reductions in mortality and hospitalization, are widely accessible, relatively low-cost, and have well-established safety profiles (McDonagh et al., 2023). Introducing a costly biologic agent that has not yet demonstrated superiority over current standard-of-care therapies may be economically unjustifiable for healthcare systems and unattractive to payers. To overcome the aforementioned barriers, robust pharmacoeconomic evidence demonstrating a favorable cost-effectiveness profile in well-defined patient subgroups would be essential, alongside advancements in molecular engineering aimed at enhancing compound stability and minimizing adverse effects.

## 9 Exploration of potential synergies with existing cardiovascular treatments

**FIGURE 5:**
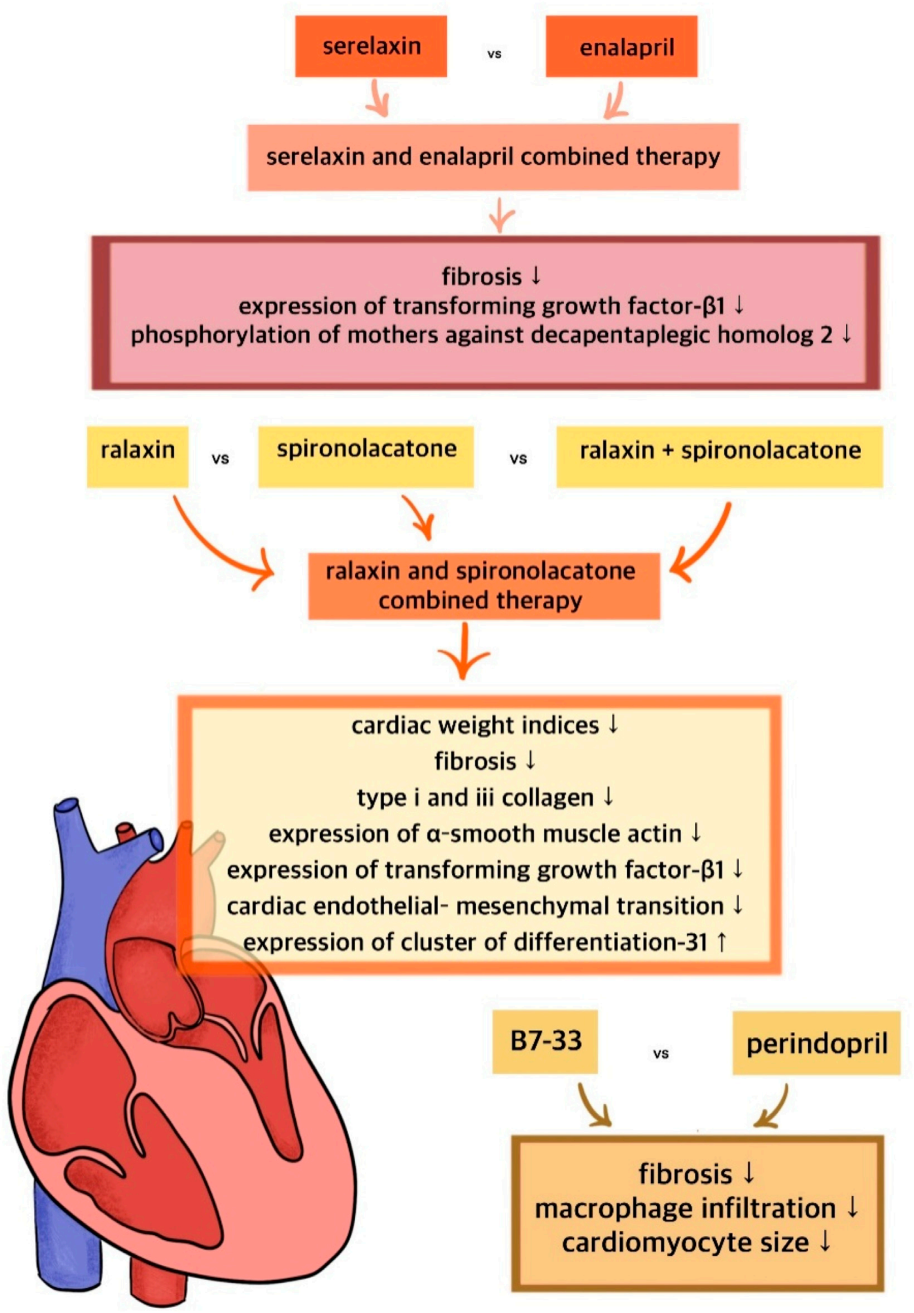
Synergism of the action of relaxin and its long-acting analogues with standard treatment.

To date, the potential synergies of long-acting relaxin analogues with other drugs have not been extensively investigated. It remains unclear whether the combination of relaxin analogues with other drugs will outperform monotherapy in terms of treatment efficacy. The combined administration of relaxin and spironolactone has been shown to significantly improve cardiac function and reduce interstitial fibrosis in isoprenaline-induced myocardial fibrosis in rats, surpassing the effects observed with the individual administration of relaxin or spironolactone (Cai et al., 2017). This finding underscores the enhanced anti-fibrotic effect resulting from the drug synergy (Cai et al., 2017). The proposed mechanism underlying this combination suggests the inhibition of cardiac endothelial-mesenchymal transition, which is believed to be primarily mediated by TGF-β (Cai et al., 2017; Piera-Velazquez and Jimenez, 2019). Additionally, B7-33 was shown to reduce TGF-β expression, further supporting the potential synergy between spironolactone and long-acting relaxin analogues (Alam et al., 2023). Given that spironolactone is a widely used and affordable drug, this combination could prove valuable in the treatment of cardiovascular diseases, including HF. The combination of relaxin and ACEI enalapril has been observed to reduce TGF-β1 expression, thereby enhancing the anti-fibrotic effect of ACEI in a murine model of cardiomyopathy (Samuel et al., 2014). The synergy may be particularly beneficial in the therapy of hypertension-related fibrosis (Samuel et al., 2014). Furthermore, the interaction between relaxin and the angiotensin type II receptor (AT_2_R) has been found to becritical for producing an antifibrotic effect (Chow et al., 2014). The MMP-promoting action of B7-33 was found to be inhibited by both RXFP1 and AT_2_R antagonist (Hossain et al., 2016). In contrast, AT_1_R blockers were shown to disrupt the anti-fibrotic action of relaxin, suggesting that the combination of relaxin analogues with sartans may reduce the efficacy of therapy and should therefore be avoided (Chow et al., 2019). The synergism of the action of the discussed molecules is shown in Figure 5.

The combination of long-acting relaxin analogs with ARNIs or SGLT2 inhibitors may offer synergistic benefits in vascular remodeling and renal protection due to their complementary mechanisms of action (Das, 2024). Relaxin exerts pleiotropic effects, including vasodilation, anti-inflammatory, and antifibrotic actions. It facilitates endothelial remodeling, enhances microvascular perfusion, and exhibits nephroprotective properties by reducing oxidative stress and improving renal hemodynamics. ARNIs, such as sacubitril/valsartan, increase endogenous natriuretic peptide levels, which - similarly to relaxin-promote vasodilation, anti-inflammatory effects, and antifibrotic activity. These actions may act synergistically with those of relaxin, particularly in reducing arterial stiffness and alleviating volume overload. SGLT2 inhibitors have well-documented nephroprotective and cardioprotective effects, including improvements in endothelial function, reductions in left ventricular filling pressures, and attenuation of inflammation and oxidative stress. Relaxin may augment these benefits through its direct protective effects on both the kidneys and the cardiovascular system. The combination of long-acting relaxin analogs with ARNI or SGLT2 inhibitors may thus provide synergistic therapeutic effects by enhancing endothelial function, mitigating fibrosis, and protecting renal and cardiac tissues. However, this approach warrants further preclinical and clinical investigation to assess safety, optimal dosing strategies, and the impact on hard clinical endpoints such as mortality and hospitalization. Further studies are needed to identify potential beneficial drug synergies, including investigating the combination of relaxin with existing HF treatments as well as new drugs such as levosimendan and proprotein convertase subtilisin/kexin 9 inhibitors (Grześk et al., 2022a; Grześk et al., 2022b).

## 10 Opportunities for further research and development

Methods to increase the efficiency of long-acting relaxin analogues are continuously being explored, as improving pharmacokinetics is crucial to maximizing their clinical utility and establishing them as the therapies of the future. Several approaches to address this issue have been proposed, although further investigation is required. Oral administration is strongly preferred over other routes of drug delivery, and achieving optimal bioavailability is essential for its efficacy. Oral bioavailability is influenced by several factors, including gut metabolism (Grześk et al., 2021). Peptides are often subject to hydrolysis and deactivation due to the potent proteolytic enzymes in the digestive system which poses a significant limitation to the use of relaxin analogues (D’Ercole et al., 2022). It is suggested that these challenges might be overcome by developing enzyme-protected pharmaceutical formulations that allow for drug release in the distal small intestine and subsequent absorption into the bloodstream (D’Ercole et al., 2022). Insulin, which shares structural similarities with relaxin, has been observed to reach therapeutic levels through oral administration when properly formulated (Praveen et al., 2023). However, orally delivered peptides face partial digestion, which can lead to an unpredictable reduction in the administered dose (D’Ercole et al., 2022).

Importantly, relaxin analogues with truncations at the chain termini have been shown to retain potent biological activity (Praveen et al., 2023). This suggests that even after partial digestion, some proteolytic fragments of relaxin analogues may, retain, to some extent, the same properties as the intact peptides (D’Ercole et al., 2022). Unfortunately, the reduction in the size of B7-33 led to a decrease in its binding affinity (Handley et al., 2023). Alternative shortened relaxin analogues such as B9-31, might be more suitable as potential therapeutic peptides (Handley et al., 2023).

The RELAX10 fusion protein, which combines relaxin-2 with an antibody Fc fragment, effectively inhibits cardiac hypertrophy and fibrosis. Moreover, RELAX10 has been shown to improve cardiac function as indicated by improvements in ejection fraction and fractional shortening (Sun et al., 2019). The effects of RELAX10 on fibrosis, hypertrophy, and cardiac function mirrored those of relaxin (Sun et al., 2019). Further research is needed to fully explore the capabilities of RELAX10. It is possible that the future of long-lasting relaxin analogues lies in lipidated peptides, which retain the effects of non-lipidated peptides while acquiring enhanced potency and metabolic stability in blood and plasma (Mallart et al., 2021). Conjugation of B7-33 with a fatty acid was found to improve its *in vitro* half-life (Praveen et al., 2023).

Another strategy to address the issue of the need for repeated doses of relaxin analogues involves the use of properly optimized portable infusion devices. Administration of relaxin via a subcutaneous infusion pump has previously been shown to result in favorable long-term outcomes in animal studies (Beiert et al., 2018; Parikh et al., 2013; Wang et al., 2016), suggesting the potential of this delivery route. Gene therapy using AAV9 vectors to upregulate RXFP1 has also shown potential in reducing myocardial infarction size (Devarakonda et al., 2022).

The development of long-acting relaxin analogs may also prove beneficial in the management of patients with atrial fibrillation (AF). Research has highlighted a connection between relaxin and molecules involved in fibrosis, inflammation, and oxidative stress in AF patients, further supporting the antifibrotic protective role of this hormone in normal human atrial cardiac fibroblasts. (NHCF-A) (Aragón-Herrera et al., 2022). In patients with AF, elevated relaxin levels were associated with a reduction in the concentrations of H2O2, mRNA of alpha-defensin 3 (DEFA3), and IL-6 in leukocytes from left atrial plasma. *In vitro* relaxin treatment inhibited normal human cardiac fibroblast (NHCF-A) migration and reduced the mRNA and protein levels of the profibrotic molecule transforming growth factor-beta 1 (TGF-β1) (Aragón-Herrera et al., 2022). In an animal model, administering 0.5 mg/kg of relaxin to rabbits for 2 weeks reduced susceptibility to AF. Mechanistically, relaxin increased the expression of peroxisome proliferator-activated receptor-gamma (PPARγ) via RXFP1 regulation, restoring mitochondrial respiration and ATP production, along with reduced reactive oxygen species in mitochondria of both rabbit atria and HL-1 cells. Furthermore, overexpression of PPARγ in tachy-stimulated HL-1 cells prevented mitochondrial damage and alleviated energy metabolism disturbances (Zhao et al., 2025). In a prospective study (n = 248), relaxin levels predicted the recurrence of AF during follow-up (with a sensitivity of 77.4% and specificity of 55.9%) in patients following radiofrequency catheter ablation (RFCA) (Qu et al., 2019). In the opinion of the authors of the presented study, a simple measurement of relaxin may help to identify patients at high risk of AF recurrence. These findings emphasize the significance of relaxin in the pathophysiology, diagnosis, and treatment of AF.

There remain numerous areas for further investigation to better understand long-acting relaxin analogues. The mechanisms of action, the processes they promote, and their affinity levels are crucial areas for future research. A comparative study of the pharmacokinetics and pharmacodynamic of various long-acting relaxin analogues is needed. It would be valuable to compare the half-life of selected long-acting relaxin analogues. Discovery programs should include a variety of concentrations of relaxin analogues to determine the most effective doses Additionally, further clarification of RXFP1 regulation in different diseases is essential for advancing further discoveries (Agoulnik et al., 2017).

## 11 Implications for personalized medicine and targeted cardiovascular therapies

Relaxin and its long–acting analogues are therapeutically relevant for patients suffering HFpEF and cardiac hypertrophy. Moreover, they have shown, in preclinical trails, to increase renal blood flow while maintaining GFR suggesting potential benefits for patients with kidney failure. Another population that may benefit from relaxin and its analogues are those with disorders in pulmonary hemodynamic adaptations (Verdino et al., 2023). Relaxin is also known for its role in the prevention of coronary thrombotic events (Dschietzig et al., 2001). The potential side effects of relaxin and its analogues areclosely related to their mechanism of action. Due to their vasodilatory effects, they may, in some cases, cause hypotension and electrolyte disturbances. Additionally, allergic reactions, particularly at the site of subcutaneous administration may occur. Gastrointestinal disorders are likely side effectof oral administration of ML290.

The potential use of relaxin in personalized therapy to meet the individual needs of patients, as well as the development of targeted therapeutic strategies for cardiovascular diseases, may lead to significant breakthroughs in treatment.

In the context of cardiovascular research, long-acting relaxin analogues show promising results in treating HF. As these therapies are futher explored, their use could potentially be considered as targeted therapy tailored to the specific needs of patients. In addition to traditional classification methods based on comorbidities, causes, left ventricular remodeling, and hemodynamic subtypes, recent advancements have combined advanced phenotyping with innovative analytical strategies, such as machine learning, to categorize HFpEF into therapeutically consistent groups, such as hypertensive HFpEF, renal HFpEF, and inflammatory HFpEF (Shah, 2017). The heterogeneous nature of HFpEF has been identified as a key factor contributing to the lack of successful outcomes in clinical trials, indicating that a one-size-fits-all approach is ineffective in treating HFpEF (Polsinelli and Shah, 2017). Consequently, future efforts will aim to optimize treatment effectiveness and direct it toward patients who are most likely to benefit from it (Tah et al., 2023).

Developing personalized therapy and tailoring it to specific patients may help reduce unnecessary side effects in individuals who may respond unfavorably to long-acting relaxin analogues. Therefore, in a recently completed clinical trial of LY3540378, the keyinclusion criteria require patients to have HF, while excluding those with a medical history of: stroke, heart transplant, cardiac arrhythmias or HF resulting from a previous pulmonary embolism. This highlights the importance of selecting the appropriate patient population for a given drug (Verdino et al., 2023).

Further research on long-acting relaxin analogues may lead to the development of new treatment options for patients with HFpEF. These therapies hold great potential to enhance treatment efficacy reduce unnecessary side effects, and personalize therapy for specifically selected target groups, maximizing the beneficial effects, such as the pleiotropic effects of LY3540378 mentioned earlier (Verdino et al., 2023).

## 12 Summary

Targeting relaxin analogues remain a significant scientific and medical challenge; however, studies havedemonstrated their promising potential as a novel therapeutic approach in cardiology, with several positive biochemical and hemodynamic effects. It remains to be determined whether long-acting relaxin analogues can replicate all the beneficial effects of natural relaxin. Furthermore, a deeper understanding of the role of relaxin, along with comparative studies assessing different long-acting relaxin analogues is essential to establish these compounds as a therapy for the future.
